# Supplementary material for: Histopathology of crustose coralline algae affected by white band and white patch diseases
Source: PeerJ. 2015 Jun 30;3:e1034. doi: 10.7717/peerj.1034 (PMC4493676; doi:10.7717/peerj.1034)
Supplement: Table S1 — HT, healthy tissue; DT, diseased tissue. [file peerj-03-1034-s001.docx]

Table S1. Type of boring organisms encountered and their localisation within the tissue for each CCA sample. HT, healthy tissue. DT, diseased tissue.

|  | Microorganisms | Type | Tissue | Layer |
| --- | --- | --- | --- | --- |
| *H. boergesenii* |  |  |  |  |
| Healthy 1 | Presence | Borer unidentified | HT | Crust |
| Healthy 2 | Presence | Sponge | DT | Limestone |
| Healthy 3 | Absence | - | - | - |
| CWBS 1 | Presence | Sponge | DT | Limestone |
| CWPD 1 | Presence | Sponge | DT | Limestone |
| CWPD 2 | Absence | - | - | - |
| CWPD 3 | Presence | Sponge | DT | Limestone |
| CWPD 4 | Absence | - | - | - |
| CWPD 5 | Presence | Sponge; Cyano | DT | Limestone |
| *N. mamillare* |  |  |  |  |
| Healthy 1 | Absence | - | - | - |
| Healthy 2 | Presence | Sponge; Borer unidentified | DT | Limestone |
| CWBS 1 | Presence | Sponge; juveniles macroborers | HT; HT | Crust and limestone; crust and limestone |
| CWBS 2 | Presence | Cyano | HT | Limestone |
| CWBS 3 | Presence | Sponge; juveniles macroborers | HT and DT; HT and DT | Crust and limestone; crust and limestone |
| CWBS 4 | Presence | Borer unidentified | DT | Limestone |
| CWPD 1 | Absence | - | - | - |
| CWPD 2 | Presence | Sponge | DT | Limestone |
| CWPD 3 | Presence | Sponge | HT and DT | Limestone |
| *P. solubile* |  |  |  |  |
| Healthy 1 | Presence | Sponge | DT | Limestone |
| CWBS 1 | Presence | Cyano | HT and DT | Limestone |
| CWBS 2 | Presence | Cyano; sponge | DT; DT | Limestone; Limestone |
| CWBS 3 | Presence | Sponge; Helminths; Borer | DT | Crust and limestone; limestone; Crust and limestone |
| *P. accretum* |  |  |  |  |
| Healthy 1 | Absence | - | - | - |
