# Supplementary material for: Histopathology of crustose coralline algae affected by white band and white patch diseases
Source: PeerJ. 2015 Jun 30;3:e1034. doi: 10.7717/peerj.1034 (PMC4493676; doi:10.7717/peerj.1034)
Supplement: Supplemental Information 2 [file peerj-03-1034-s002.docx]

**Appendix**

Sharman staining procedure (Sharman 1943) modified from Ruzin (1999)

**Procedure**

Tissues may be preserved with any fixative.

1. Deparaffinize in Limonene (3 baths of 3 minutes each), followed by hydration in a graded EtOH series (2 baths x 3 min in 100%, 2 baths x 3 min in 70%)
2. Transfer for 1 min to filtered 2% ZnCl2 (aq).
3. Wash in DI for 5 s.
4. Stain for 5 min in Safranin O staining solution.

*Staining*

1. Wash in DI for 5 s.
2. Transfer to Orange G staining solution and stain for 1 minute.
3. Wash in DI for 5 s.
4. Transfer to tannic acid solution for 5 min.
5. Wash in DI for 1–3 s.
6. Transfer to 1% aq iron alum [(NH4Fe(SO4)2, filtered] for 2 min.
7. Wash in DI for 5–15 s.
8. Dehydrate through 45, 90, 100% EtOH, about 10 s each step.
9. Transfer to Limonene (three baths of three minutes each)

Orange G staining solution:

Orange G 2 g Tannic acid 5 g HCl (conc) 4 drops DI to 100 ml

Add thymol, phenol, or azide (0.03% w/v) to inhibit microorganism contamination. Azide can be used at 0.03–0.1% w/

Filter before use

Tannic acid solution:

Tannic acid 5 g DI to 100 ml

Add thymol, phenol, or azide (0.03% w/v) to inhibit microorganism contamination

Filter before use

Safranin O stock solution:

Safranin O 1 g DI 50 ml

Safranin O staining solution:

Safranin stock (2%) 1–1.5 ml DI 500 ml

References:

Ruzin (1999). Plant Microtechnique and Microscopy
